# Is peres formula reliable for determination of proper position of central venous catheter tip in Iranian population?

**DOI:** 10.34172/jcvtr.025.33436

**Published:** 2025-06-28

**Authors:** Maryam Ghanbari Garekani, Amirhossein Poopak, Hamidreza Pouraliakbar, Arash Barghi, Razieh Omidvar, Ziae Totonchi

**Affiliations:** ^1^Rajaie Cardiovascular Medical and Research Center, Iran University of Medical Sciences, Tehran, Iran; ^2^Heart Valve Disease Research Center, Rajaie Cardiovascular Medical and Research Center, Iran University of Medical Sciences, Tehran, Iran

**Keywords:** Peres formula, Central venous catheter tip depth, Anatomical variations, Body mass index and height correlations

## Abstract

**Introduction:**

Accurate positioning of central venous catheter (CVC) tips is essential to minimize complications such as arrhythmias, thrombosis, or cardiac tamponade.

**Methods:**

This study evaluated the reliability of the Peres formula, which estimates CVC tip placement based on patient height, within an Iranian population. A cross-sectional analysis of 100 patients undergoing cardiac surgery revealed that the Peres formula often resulted in incorrect CVC positioning, necessitating radiographic confirmation and post-insertion adjustments.

**Results:**

The mean deviation of CVC tip placement from the ideal position near the carina was 5.13±0.78 cm. Correlation analysis highlighted significant associations between the deviation and demographic factors, including height and body mass index (BMI), suggesting the need for population-specific adjustments to the Peres formula.

**Conclusion:**

These findings underscore the importance of tailored approaches to CVC placement to account for anatomical and physiological differences, emphasizing the need for modified guidelines for the Iranian population to enhance safety and accuracy in clinical practice due to the fact that Peres formula is not suitable for Iranian population.

## Introduction

 Central Venous Catheterization (CVC) is an invasive but vital procedure used in surgical operations, especially among ill patients, for maintaining volume conservation, blood transfusion, hemodialysis, chemotherapy, and antibiotic therapy.^1^ The textbook excerpt lists indications for CVC placement, including rapid fluid infusion, frequent blood sampling, and venous access to vasoactive drugs. It also highlights the risk of air embolism and contraindications like pre-existing venous obstructions or severe coagulopathies, further emphasizing the importance of accurate CVC placement. Incorrect CVC is accompanied by some side effects such as pneumothorax, carotid artery penetration and hematoma, cardiac arrhythmia, thrombosis, and infection.^2-4^ Although the standard location of CVC insertion is the connection of the right atrium to the superior vena cava, traditionally, some procedures, including superficial and anatomical markers and paraclinical procedures such as electrocardiogram changes, post insertion radiological study, and transesophageal echocardiography had been used for determination of the proper location of CVC.^5^

 In most patients needing CVC insertion, post-insertion chest radiography is used to determine the correct location of CVC tips according to carina bifurcation place as a radiological marker of the right atrial and superior vena cava connection place. Chest radiography was time-consuming and cannot be used during the CVC insertion procedure. Moreover, fluoroscopy during characterization was suggested in some cases to determine the right CVC location. Fluoroscopy is expensive, has the risk of X-ray radiation, and cannot be performed in all situations. Finally, Transesophageal echocardiography, as the latest suggestion for this issue, is expensive and needs an expert operator to perform.^6-9^

 Peres formula is suggested to confirm the right location of CVC in the right and left internal jugular veins and right and left subclavian veins; it is a simple and memorable formula. However, in some cases, CVC was entered into the right atrium and led to arrhythmia and even cardiac tamponade.^10,11^ Moreover, racial and anatomical differences can impact formula calculation and incorrection CVC insertion occurs. For instance, three-centimetre corrections were suggested for using the Peres formula among Indonesian patients ^11^. Unfortunately, we did not do any study using the Peres formula among Iranian patients with different racial and physical situations compared to other patients. The present study was performed to confirm the result of the Peres formula calculation using the standard method among Iranian patients referred to Shahid Rajaie cardiovascular institute.

## Materials and Methods

 A present cross-sectional study was performed on One hundred patients between the ages of 18 and 70 who underwent open heart surgery and a central venous catheter was inserted through the right internal jugular vein. Cardiac surgery in patients included these items: CABG, valvular repair or replacement, myotomy, aneurysm repair, and congenital heart disorders.

 According to the census sampling method, the study population was selected from all patients who needed CVC insertion during the study period. Among included patients, those with a history of previous neck surgery, abnormal neck anatomy, local neck infection, chest deformities, pacemakers, coagulopathy and patients who expired in operating room were excluded.

**Figure 1:**
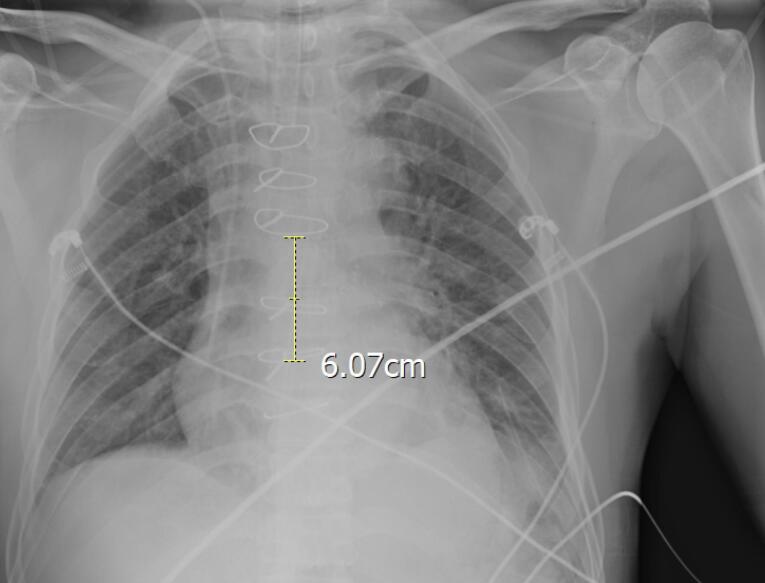
chest radiography of study patients with incorrect CVC tip insertion according to Peres formula

**Figure 2:**
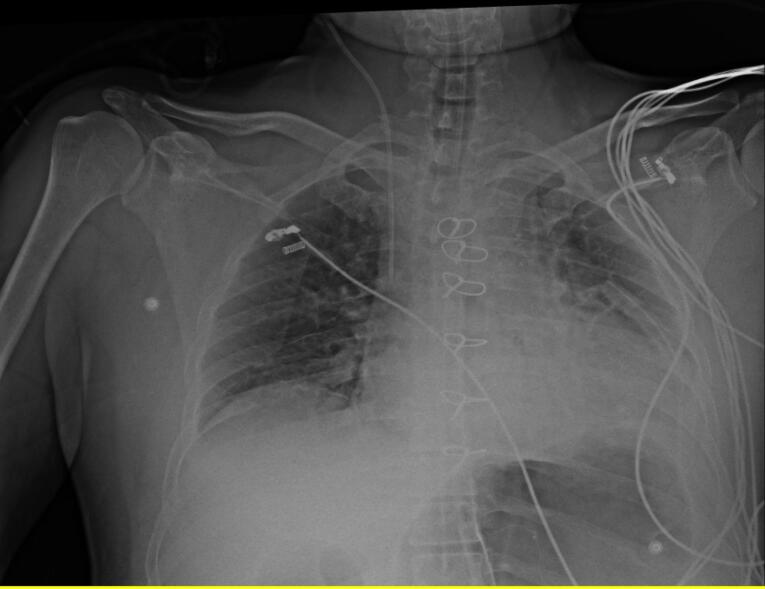
landmarking of correct CVC tip location according to carina in patient with incorrect CVC tip position according to Peres formula

**Figure 3:**
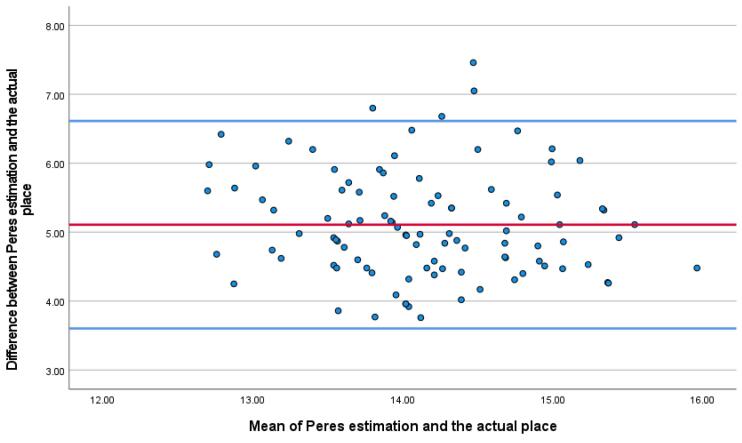
Bland-Altman plot comparing the Peres estimation and the actual place of the CVC tip

 After preliminary checks, demographic data including Age, gender, weight (kg), height (cm), and body mass index (BMI) (kg/m^2^) were gathered. All 100 CVC were inserted in the internal jugular vein with disinfection method with alcoholic chlorhexidine antiseptic for skin preparation, then cvc was fixed according to the Press formula by a cardiac anesthesiologist in the operating room (OR). According to Peres’ formula, the depth of CVC insertion should be height/10 cm for the right internal jugular vein (IJV), after fixation Chest radiography was performed for patients in the supine position, and the tip position of the catheter was assessed according to the distance between the CVC tip and the carina. We measured the distance between the carina and CVC and recorded it as a Distance variable. One centimeter distance from the carina (higher and lower) is the proper CVC tip position, and other patients were in the wrong CVC tip position and cvc was fixed in correct depth. All of the study demographic and variables related to surgery accompanied by the result of chest radiography were gathered and inserted into the patient list. Figure 1 shows the chest radiography of one patient with incorrect CVC tip insertion. Figure 2 landmarked the proper position of CVC tips according to the carina location on the chest radiography. Figure 3 shows the Bland Altman plot for the Peres estimation and the actual place. The x-axis represents the mean of the Peres estimation and the actual place, while the y-axis shows the difference between the two. The plot includes a red line indicating the mean difference (or bias) between the estimations and actual values, with the blue lines representing the 95% limits of agreement (mean difference ± 1.96 standard deviations). Each point represents an individual data pair, illustrating the variability between the estimation and actual place across different values. This plot is used to assess the agreement between the two methods.

###  Statistical analysis

 Study data were gathered and inserted into the SPSS version 24.0 (IBM Corp., Armonk, NY, USA) package for analysis. Mean/standard deviation and frequency/percentage were used to describe quantitative and qualitative variables. Chi-square, independent t-tests and bland altman were used to compare qualitative and quantitative variables between study groups, respectively. All P-values less than 0.05 were assumed to be significant results.

## Results

 Finally, 100 patients were entered into the analysis; 69 (69%) were male and 31 (31%) were female. Study participants’ mean age and BMI were 55.63 ± 12.16 years and 27.13 ± 4.63 kg/M^2^, respectively. (Table 1)

**Table 1:** Correlation analysis of quantitative variables with Distance variable

	**Distance**	**Age**	**height**	**Weight**	**BMI**
Distance	-	δ = -0.13	δ = 0.37	δ = -0.02	δ = -0.21
	-	P = 0.19	P 0.001	P = 0.82	P = 0.04
Age	δ = -0.13	-	δ = -0.018	δ = 0.15	δ = 0.11
	P = 0.19	-	P = 0.06	P = 0.88	P = 0.29
Height	δ = 0.37	δ = -0.018	-	δ = 0.24	δ = -0.25
	P 0.001	P = 0.06	-	P = 0.02	P = 0.02
Weight	δ = -0.02	δ = 0.15	δ = 0.24	-	δ = 0.85
	P = 0.82	P = 0.88	P = 0.02	-	P 0.001
BMI	δ = -0.21	δ = 0.11	δ = -0.25	δ = 0.85	-
	P = 0.04	P = 0.29	P = 0.02	P 0.001	-

 According to findings of chest radiography of study participants, CVC had incorrect tip position in all of the patients and needed correction by measuring distance at chest radiography. The mean distance between CVC and carina was 5.1268 ± 0.7784 (3.74-7.46) cm. The Mean CVC distance difference was insignificant between male and female patients (5.21 ± 0.78 vs. 4.94 ± 0.76; *P* = 0.12). In the correlation analysis, the study participants’ height and BMI significantly correlated with the CVC distance variable. Details of the correlations are presented in Table 2.

**Table 2:** Comparison mean of CVC distance variable among different cardiac operations

**Operation types**	**Frequency**	**Mean**	**Standard Deviation**
CABG	54	5.11	0.69
Valvar repair or replacement	38	5.18	0.82
Myotomy	3	4.78	1.27
Aneurism repair	1	7.050	-
Congenital heart disorders	4	4.67	0.544
Total	100	5.13	0.78
*P*-value^*^		0.08	

*Calculated by analysis of variance statistical test

 The mean distance between the carina and CVC tip was not significantly different between the different surgical operations performed on study participants.

## Discussion

 The central placement of venous catheters (CVCs) is critical in managing critically ill patients requiring long-term intravenous therapies or hemodynamic monitoring. However, the precise position of the catheter tip is vital to avoid complications such as thrombosis, arrhythmia, or cardiac tamponade, pneumothorax, cardiac arrhythmia, thrombosis, and infection.The Peres formula (Height/10 cm for the right internal jugular vein) is often used to estimate the correct CVC tip position. However, its reliability varies based on racial and anatomical differences. While effective traditional methods such as chest radiography present logistical and radiological challenges, this study aimed to validate the Peres’ height formula as a reliable tool for the placement of CVCs among the Iranian population, potentially offering a more straightforward and radiation-free alternative.

 Our results indicate that despite its simplicity and widespread use in certain regions, the Peres formula needs to be re-examined regarding the Iranian population according this cohort study. The study highlights discrepancies in the Iranian population when using the Peres formula, often leading to incorrect CVC placement. Chest radiography revealed a mean deviation of 5.13 ± 0.78 cm between the tip and the carina, requiring post-insertion corrections. These findings suggest that anatomical and physiological differences (e.g., height, BMI, thoracic structure) significantly affect CVC placement accuracy. This finding contrasts with previous studies conducted in different ethnic groups, where the Peres formula showed higher accuracy.^11^ These variations highlight the influence of genetic and regional anatomical differences on medical formulae initially developed in other populations.

 Misplaced catheters can lead to severe complications, including arrhythmias or cardiac tamponade. Thus, while the Peres formula provides a quick estimation, its limitations underscore the importance of radiographic confirmation post-insertion.

 Traditional radiographic methods (e.g., chest X-rays, fluoroscopy) and advanced techniques like transesophageal echocardiography provide higher accuracy but are time-consuming, expensive, and require skilled operators. The study findings suggest modifying the Peres formula to consider regional anatomical variations, such as incorporating BMI and height. Interestingly, while the mean distance between the CVC tip and the carina was uniform across genders, significant correlations were observed with the patient’s height and BMI. This suggests that while Peres’ formula considers height, other factors, such as BMI and perhaps thoracic anatomy variations, influence the ideal catheter length. Similar studies have adjusted the formula for specific populations, indicating a potential need for a tailored approach in the Iranian population.^12^

 The lack of significant variation in CVC tip placement across different types of surgical interventions also suggests that the inaccuracies of the Peres formula are consistent regardless of the procedure type. This consistency further underscores the potential anatomical and procedural factors influencing CVC placement that are not accounted for by the formula.

 Given these findings, it is imperative to consider modifying the Peres formula for the Iranian population. One approach could be integrating BMI alongside height in the formula, which our data suggest could enhance accuracy. Additionally, a more extensive, multi-center study could provide the data necessary to develop a modified formula that better suits different Iranian sub-populations anatomical and physiological profiles. We recommend changing the Peres formula for the Iranian population for further investigations, potentially including variables like BMI. A more extensive, multi-center study is required to validate the formula adjustments.

## Conclusion

 In conclusion, while the Peres formula provides a valuable starting point for estimating CVC tip position, it cannot be relied upon solely in its current form to ensure accurate placement within the Iranian population. The standard practice of post-insertion radiography remains indispensable and should continue until a more reliable and universally applicable method is developed. Further research into tailored formula modifications or alternative non-radiative techniques, such as intravascular ECG, could provide safer and more efficient methods for CVC placement in diverse patient populations.

## Competing Interests

 Non to declare.

## Ethical Approval

 The study protocol was approved by the research ethical committee of Rajaie cardiovascular Medicine and Research Institute IR.RHC.REC.1403.051 and all signed informed consent.
